# Continuous Hydrothermal Flow Synthesis and Characterization of ZrO_2_ Nanoparticles Doped with CeO_2_ in Supercritical Water

**DOI:** 10.3390/nano12040668

**Published:** 2022-02-17

**Authors:** Qingyun Li, Lingyu Liu, Zihua Wang, Xuezhong Wang

**Affiliations:** 1 School of Chemistry and Chemical Engineering, South China University of Technology, Guangzhou 510641, China; 201610103635@mail.scut.edu.cn (Q.L.); lly@xcu.edu.cn (L.L.); 2 Pharmaceutical and Crystallization Systems Engineering Group, Beijing Key Laboratory of Enze Biomass and Fine Chemicals, College of New Materials and Chemical Engineering, Beijing Institute of Petrochemical Technology, Beijing 102617, China

**Keywords:** continuous hydrothermal flow synthesis, confined jet mixing reactor, CeO_2_-ZrO_2_ nanoparticles, XRD, BET

## Abstract

A confined jet mixing reactor operated in continuous hydrothermal flow synthesis was investigated for the synthesis of CeO_2_-ZrO_2_ (CZ) nanoparticles. The obtained ultrafine powders were characterized using scanning electron microscopy–energy dispersive spectrometry (SEM-EDS), inductively coupled plasma–atomic emission spectroscopy (ICP-AES), Fourier transform infrared spectroscopy (FTIR), Raman spectroscopy, X-ray diffraction analysis (XRD), transmission electron microscopy (TEM) coupled with selected area electron diffraction (SAED), a BET (Brunauer-Emmett-Teller)-specific surface area test and pore analysis, oxygen storage capacity (OSC) test, and a H_2_ temperature programmed reduction (H_2_-TPR) test. The XRD results show that all samples were composed of high-purity cubic CZ nanoparticles. High resolution transmission electron microscope (HR-TEM) analysis showed that CZ nanoparticles with uniform size and shape distributions were obtained in this investigation. The d-spacing values, determined based on the TEM-selected area electron diffraction (SAED) patterns, were in good agreements with the reference data. BET results showed that the prepared CZ samples had large specific surface areas. Pore volume and size distribution were obtained by pore analysis. Oxygen pulse adsorption technology was used to test the oxygen storage capacity of the sample. The redox capacity of the CZ material was determined by a H_2_ temperature-programmed reduction test.

## 1. Introduction

CeO_2_-ZrO_2_ (CZ) with a cubic structure has been extensively investigated for catalysis [1], oxygen storage/release capacity (OSC) [2], gas sensors [3,4,5], solid oxide fuel cells (SOFCs) [6,7], and thermal insulation [8] due to its excellent physical and chemical properties, such as high surface area [9,10], good thermal stability [2,6,11], and excellent electrochemical performance [12,13].

The traditional methods developed for producing CZ, such as hydrolysis [14], precipitation [15], citrate complex [16], etc., are all time consuming and involve complex post-processing, organic solvent use and high energy consumption. Continuous hydrothermal flow synthesis (CHFS) technology, as a simple, efficient and environmentally friendly nanomaterial synthesis process, has attracted considerable attention in recent years [17,18,19,20]. This process involves the mixing of one aqueous metal salt solution with a supercritical water (SCW) stream within a continuous reactor to produce nanosized metal oxide particles. Compared to the other synthesis methods, CHFS has shown the advantages of a fast reaction rate, short residence time, and independent parameter control, such as pressure, temperature, and concentration.

The CHFS of inorganic nanoparticles, using SCW as a reaction medium and heat source which is environmentally friendly, offers many advantages over batch and other synthesis processes. Firstly, it is easy to adjust and control the reaction parameters such as pressure, temperature, concentration, reaction time, etc., for the purpose of controlling the particle size, morphology, and surface properties of products [21]. Secondly, CHFS has the ability to produce a range of nanoparticle compositions in a short time and guarantee the product quality consistency. Thirdly, the products of CHFS usually have high crystallinity, narrow particle size distribution (PSD), uniform particle shape and extra fine particle size [22]. When a flow of aqueous metal salt is mixed with supercritical water (374 °C and 22.1 MPa), the rapid hydrolysis of the metal salt occurs in the mixture immediately followed by dehydration, thus forming the oxide. The rapid nucleation of nanoparticles is facilitated by the change in the properties (density, dielectric constant, ion product) of water under supercritical conditions, which affects the solubility of species in solution, thus forcing precipitation. The short reaction time (a few seconds) limits the growth and aggregation of particles, which in turn promote the production of ultra-fine particles [23].

Instead of slowly heating the solutions, the CHFS system can reach an extremely high temperature within seconds. This is achieved by mixing the aqueous precursor solution with a stream of supercritical water. In this way, the formulation temperature is raised to a near or above-critical level, and the time spent in the heating-up period is minimized. As the temperature increases, the rate of hydrothermal reaction is accelerated to a great extent, the degree of supersaturation increases, and highly crystallized nanoparticles form [24,25].

Kim et al. [26,27] prepared a ceria–zirconia complex metal oxide using continuous hydrothermal synthesis in supercritical water, and the catalytic capacity of the material was measured. Weng et al. [28] developed a novel high-throughput continuous hydrothermal (HiTCH) flow synthesis system with a countercurrent mixer (CCM) reactor that can be used to directly and rapidly generate a 66-sample nanoparticle library for nanocrystalline Ce_x_Zr_y_Y_z_O_2−δ_ in less than 12 h. Weng et al. [29] synthesized two Ce–Zr–La–Y oxide solid solutions with different compositions through an analogous continuous hydrothermal process.

**Figure 1:**
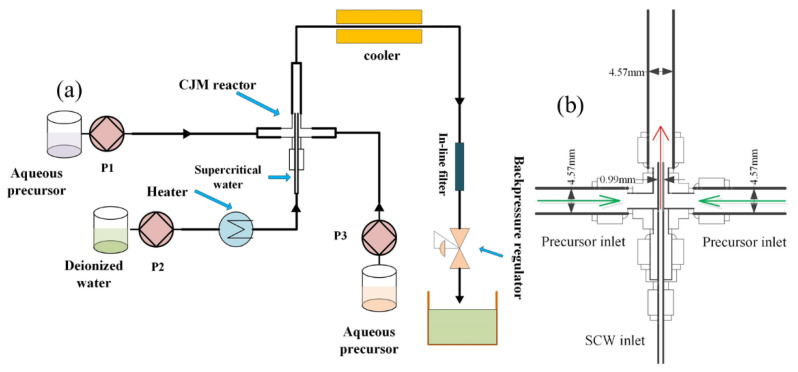
(**a**) Flow diagram of the CHFS system and (**b**) a schematic diagram of the CJM reactor.

In this work, a confined jet mixer (CJM) was employed in a CHFS system (Figure 1). The mixing study in our previous work found that CJM offers excellent effective mixing between supercritical water and precursor streams [30,31]. Gruar et al. [32] carried out research to scale up CJM for the continuous hydrothermal manufacture of ZnO_2_ nanomaterials.

The objective of the current study was to synthesize high-CZ nanoparticles with crystallinity and a narrow size distribution. To clarify the specific features of the CHFS system, the temperature, pressure, and concentration of the precursor solution as well as the cerium doping amount were investigated. Characterization was carried out using XRD, FTIR, RAMAN spectroscopy and TEM. The specific surface area and pore structure of CeO_2_-ZrO_2_ nanoparticles were analyzed using BET, t-plot and BJH (Barrett-Joyner-Halenda) analysis. The oxygen storage and reduction properties of CeO_2_-ZrO_2_ nanoparticles were characterized by means of an oxygen storage capacity (OSC) test and H_2_ temperature-programmed reduction test (H_2_-TPR), and the effect of Ce doping on the properties of binary mixed oxides was analyzed.

## 2. Materials and Methods

### 2.1. Materials

Pure zirconyl nitrate [ZrO(NO_3_)_2_·xH_2_O (≥99.5% purity)] and cerium nitrate hexahydrate [CeN_3_O_9_·6H_2_O (99.95% purity)] were used as starting materials. The starting solutions were prepared by dissolving metal salts into distilled water. The pH was adjusted by adding KOH (99.9% purity) solution for all experimental runs. All chemical reagents were obtained from Aladdin Bio-Chem Technology Co., Ltd. (Shanghai, China).

### 2.2. CHFS System

The experimental apparatus is shown in Figure 1a. Deionized water was heated to 723 K and pumped into the CJM reactor (Figure 1b) by pump P2 at a constant flow rate of 10 mL/min. According to different proportions, appropriate amounts of ZrO(NO_3_)_2_·xH_2_O and Ce(NO_3_)_3_·6H_2_O were prepared in a 1000 mL precursor salt solution. A stream of aqueous precursor solution (0.1 M, pumping rate at 5 mL/min) was pumped into the CJM reactor using pump P1, and a flow of KOH solution (0.05 M, pumping rate at 5 mL/min) was pumped through P3 at room temperature. To maintain the temperature of the mixing point, an auxiliary heating unit was wrapped around the CJM reactor. The system pressure was maintained at 24 ± 0.1 MPa. The obtained nanoparticles travelled upwards for rapid cooling and then passed through a 7 μm filter to remove large aggregates before harvesting. Then, the collected aqueous suspension was separated by centrifugation and washed in distilled water. The damp solid was then dried in an oven at 343 K for 24 h. The preparation ratio of the precursor salt solution is listed in Table 1.

### 2.3. CZ Characterization

#### 2.3.1. SEM-EDS

In this paper, a Zesis Merlin scanning electron microscopy–energy dispersive spectrometer was used to characterize the composition of CZ samples. The powder samples were directly attached to a conductive gel sample stage and gold-sprayed prior to the test.

#### 2.3.2. ICP-AES

Inductively coupled plasma–atomic emission spectroscopy is mainly used for elemental content analysis. In this paper, the ICP-AES (PerkinElmer Optima 8300, Waltham, MA, USA) characterization test was used to determine the content of metal elements in the prepared nanoparticles, and the samples were nitrated by aqua regia.

#### 2.3.3. X-ray Diffraction

The crystal phase of the prepared CZ samples was further confirmed by X-ray diffraction (X’pert Powder, PANalytical, Holland) with a PSD detector over a scattering angle (2θ) range of 5° to 90° using Cu Kα radiation generated at 40 mA and 40 kV at a scanning rate of 0.02°/s.

#### 2.3.4. FTIR

The FTIR spectra were recorded using a Nicolet IS50 Fourier transform infrared spectrometer over the spectral range from 4000 to 400 cm^−1^ at a resolution of 1 cm^−1^ with a mirror velocity of 0.88 cm/s, leading to modulation frequencies in the range of 70–704 Hz.

#### 2.3.5. RAMAN

The Raman spectra were obtained with a HJY LabRAM Aramis laser Raman spectrometer at room temperature. A 785 nm laser in a backscattering configuration was used to excite the samples. To avoid sample heating, a laser power of less than 50 mW was used.

#### 2.3.6. TEM-SEAD

The size and morphology and elemental composition of the particles were characterized using transmission electron microscopy (TEM; JEOL JEM-2100F, Japan) and selected area electron diffraction (SAED, Japan). The TEM specimens were prepared by dispersing the dried powder into acetone, followed by drop casting onto a microgrid.

#### 2.3.7. BET-Specific Surface Area Test

The BET-specific surface area of the samples was measured by an MAC-specific surface area and pore size distribution tester (ASP-2010, USA). The samples were degassed at 120 °C for 4 h in vacuum and measured at 77 K with liquid nitrogen as the adsorbent.

#### 2.3.8. Oxygen Storage Capacity (OSC) Test

The OSC of the samples was measured by using oxygen pulse adsorption technology with TPD/TPR (AutoChem1 II 2920, USA). The test procedure was as follows: the sample was reduced at 550 °C for 40 min with pure H_2_ and then purged with deoxygenated high-purity N_2_ (flow rate: 40 mL/min) until the sample temperature dropped to 200 °C. After the baseline for the recorder became straightened, oxygen was pulsed every 5 min at a constant temperature (flow rate: 30 mL/min) to calculate the adsorption volume of O_2_.

#### 2.3.9. H_2_-TPR Test

The H_2_ temperature programmed reduction test device TPD/TPR (AutoChem1 II 2920, USA) was used to measure the redox capacity of the materials. The samples were weighed and placed into a quartz reaction tube at 400 °C, pretreated in a N_2_ atmosphere for 30 min, cooled to room temperature, and switched to a N_2_ (90%)-H_2_ (10%) mixture with a flow rate of 40 mL/min. After the baseline became flat, temperature-programmed reduction was carried out at a heating rate of 10 °C/min and detected using a thermal conductivity detector (TCD, USA).

## 3. Results and Discussion

### 3.1. Elemental Analysis

**Figure 2:**
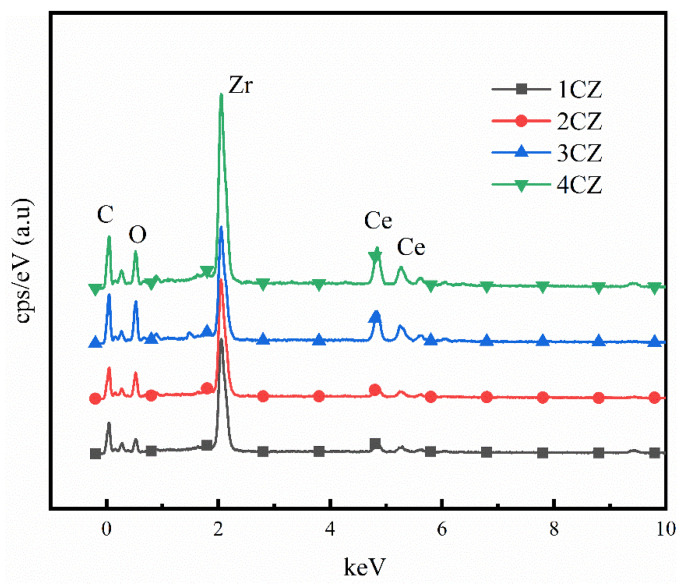
SEM-EDS spectra of the CZ nanoparticles obtained under various operating conditions.

SEM-EDS energy spectrum (Figure 2) and ICP-AES were used to characterize and test the chemical composition and metal element content of the samples prepared. Four CZ samples were prepared with high purity. Note that the element C was produced by the carbon contained in the conductive adhesive during the preparation process. The stoichiometric ratio of CZ samples obtained by analysis was close to the initial set ratio, as shown in Table 2.

### 3.2. X-ray Diffraction

**Figure 3:**
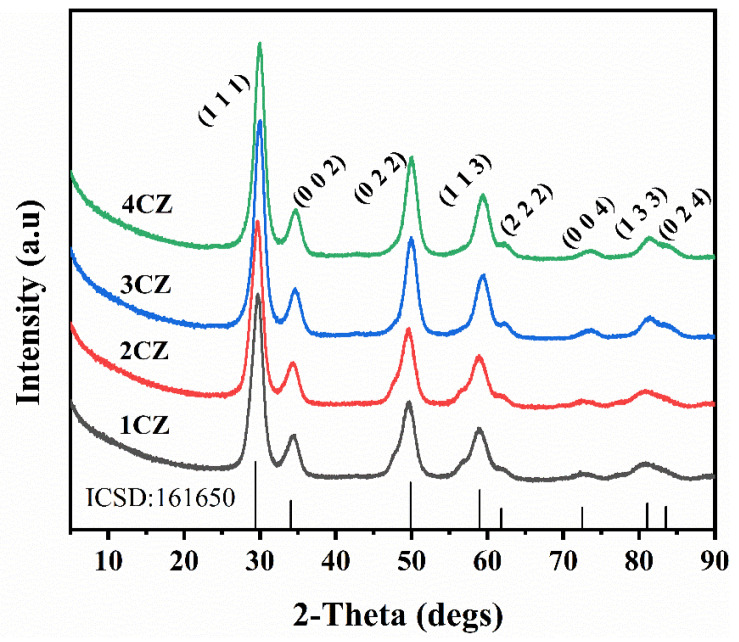
XRD analysis of the CZ nanoparticles.

As shown in Figure 3, all samples can be entirely indexed as cubic phase (ICSD 161650) CZ crystals labelled at the top of each peak. No extra peaks were found, indicating that the CZ nanoparticles were of high purity. Wide peak broadenings suggest that these are ultrafine nanoparticles [33]. At the same time, it can be observed that no diffraction peaks belonging to the monoclinic phase and tetragonal phase ZrO_2_ were found in the diffraction curves for all CZ crystals. In addition, no diffraction peak belonging to CeO_2_ was found in the diffraction patterns measured for all samples, which also indicates that Ce entered the ZrO_2_ lattice.

The mean crystallite size of CZ nanoparticles was calculated using the Scherrer formula [34]:

(1) $$D=\frac{\kappa \lambda }{\beta \cos\theta }$$

where *κ* is the shape factor and has a typical value of 0.89, *λ* is the wavelength of the X-rays (Cu *κ*, *λ* = 0.15405 nm), *β* is the full width at half maximum (FWHM) of the most intense peak (convert it to radians in the process of calculation), and *θ* is the peak position. The calculated average crystallite sizes of the synthesized particles were found to be approximately 10 nm.

### 3.3. FTIR

**Figure 4:**
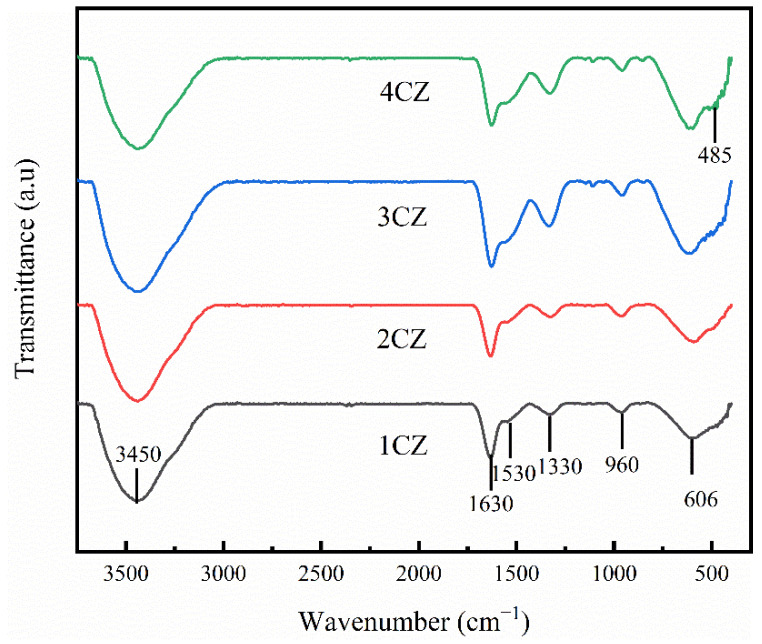
FTIR spectra for the CZ nanoparticles.

The FTIR spectra for the obtained CZ samples are illustrated in Figure 4. The broad band centered at approximately 3450 cm^−1^ could be a result of the atmospheric moisture retained by the KBr pellet and from the stretching frequencies of −OH groups in the absorbed water [35]. The band at 1630 cm^−1^ can be attributed to coordinated water due to the “scissor” bending mode of molecular water [35,36]. The absorption peak at approximately 1530 cm^−1^ is due to the vibration of the Zr−OH bond [37]. The peak centered at approximately 1330 cm^−1^ can be associated with stretching vibrations of the Zr−O terminal [38]. The peaks observed at approximately 960 cm^−1^ and 606 cm^−1^ are characteristic absorption peaks of cubic zirconia (c-ZrO_2_) [37,38,39,40]. There is a weak absorption peak in the 485 cm^−1^ wavenumber region of the infrared spectra for 3CZ and 4CZ, which may be due to absorption by a small amount of tetragonal zirconia crystals. However, no obvious absorption peak was observed in the wavenumber region below 500 cm^−1^ in the infrared spectra for 1CZ and 2CZ.

### 3.4. RAMAN

**Figure 5:**
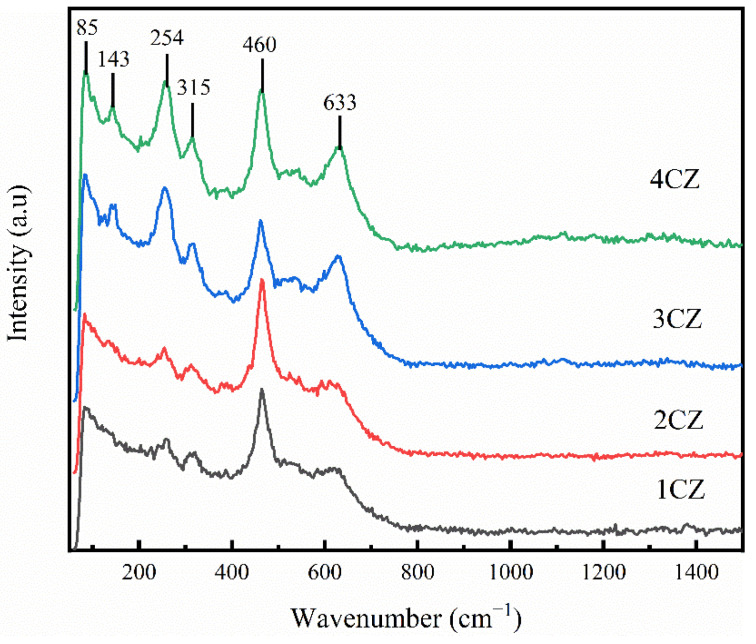
Raman spectra for the CZ nanoparticles.

To further confirm the phase composition of the CZ nanoparticles, Raman spectra were used to identify the crystal structure of representative samples. As shown in Figure 5, the absorption peaks for CZ samples at 85 cm^−1^, 143 cm^−1^, 254 cm^−1^, 315 cm^−1^ and 633 cm^−1^ are characteristic absorption peaks of tetragonal zirconia (t-ZrO_2_), and 460 cm^−1^ is a characteristic absorption peak of cubic zirconia (c-ZrO_2_) [41]. There was no obvious strong absorption peak at 143 cm^−1^ for 1CZ and 2CZ, and the absorption peaks at 254 cm^−1^, 315 cm^−1^ and 633 cm^−1^ were weak. However, there was an obvious strong absorption peak at 460 cm^−1^, which is a characteristic peak of the cubic phase, indicating that the main structure of the 1CZ and 2CZ crystals was the cubic phase, but there was only a small quantity of tetragonal phase crystals. As shown in Figure 5, with increased Ce doping, the characteristic peaks for the tetragonal crystals were enhanced, indicating that the amount of tetragonal crystals gradually increased. However, there were no characteristic peaks for monoclinic crystals in the XRD, IR and Raman spectra, indicating that ZrO_2_ doping with Ce^4+^ is conducive to improving the stability of t-ZrO_2_ and c-ZrO_2_.

### 3.5. TEM-SEAD

**Figure 6:**
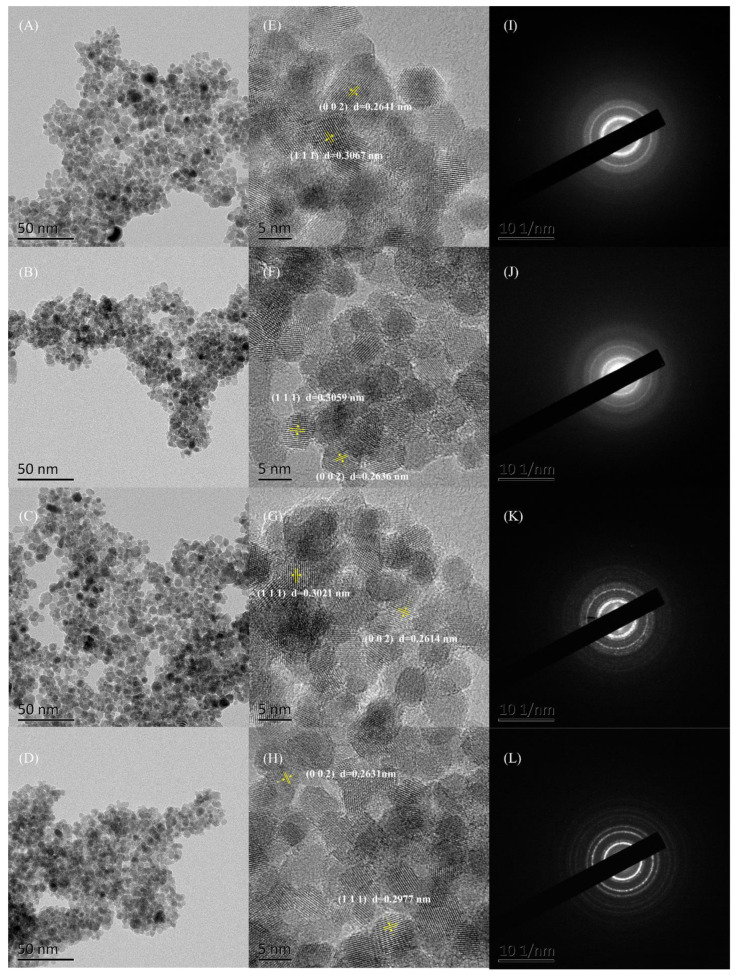
(**A**–**D**) TEM images, (**E**–**H**) HR-TEM images and (**I**–**L**) SAED patterns for the CZ nanoparticles.

The morphology of the obtained powders was investigated using transmission electron microscopy (TEM). As shown in Figure 6, the numbers for (A)–(D) represent 1CZ, 2CZ, 3CZ, and 4CZ, respectively. As observed in Figure 6, highly crystalline CZ was observed in all obtained samples, showing relatively uniform shapes with narrow size distributions. According to the ICSD 161650 standard card data, 2θ = 29.25° and 33.91° correspond to the (1 1 1) and (0 0 2) crystal planes, respectively, and the lattice spacings were d = 0.3051 nm and 0.2642 nm, respectively. The D-spacings of the (1 1 1) and (0 0 2) planes measured by HRTEM (shown in Figure 6E–H) were in good agreement with the reference data [ICSD 161650]. Most of the grains in the figure have parallel and equidistant lattice fringes, without a lattice mismatch, indicating that the sample crystallizes well, which is consistent with the selected area electron diffraction (SAED) results shown in Figure 6I–L.

### 3.6. Specific Surface and Pore Analysis

**Figure 7:**
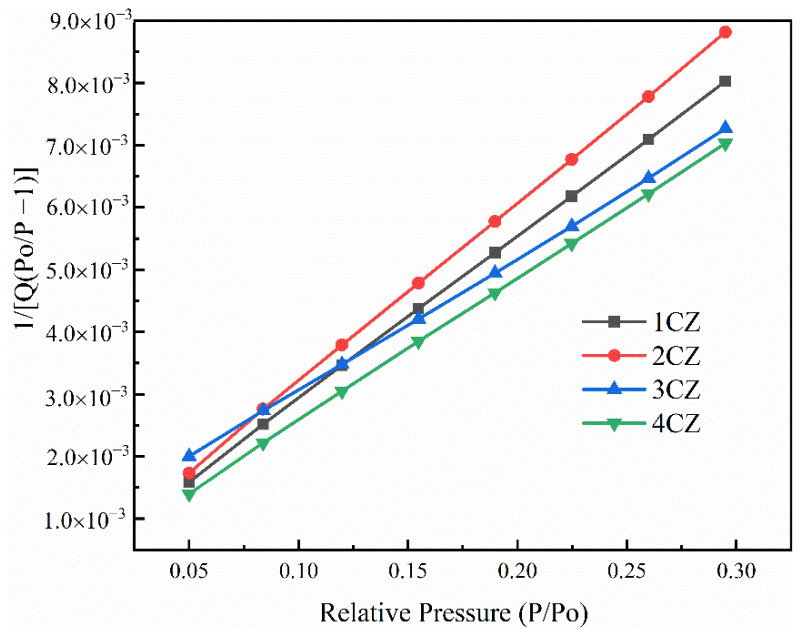
BET surface area plot for the CZ nanoparticles.

The BET-specific surface area of the CZ sample was degassed in vacuum for 4 h at 120 °C and at 77 K with liquid nitrogen as the adsorbent, as shown in Figure 7. The BET-specific surface area, constant C and regression coefficient for the four groups of CZ samples are listed in Table 3. According to the data in Table 3, the C values for all samples ranged between 50 and 200, which conforms to the characteristics of the C values of oxides. The regression coefficients were all above 0.9999, indicating that the adsorption lines for the samples in the range of P/Po = 0.05~0.35 were in good agreement with the BET equation, and the test data values were close to the actual surface area. As given in Table 3, the CZ samples all show large specific surface areas, while the addition of Ce has no clear effect on the specific surface area of the CZ samples.

**Figure 8:**
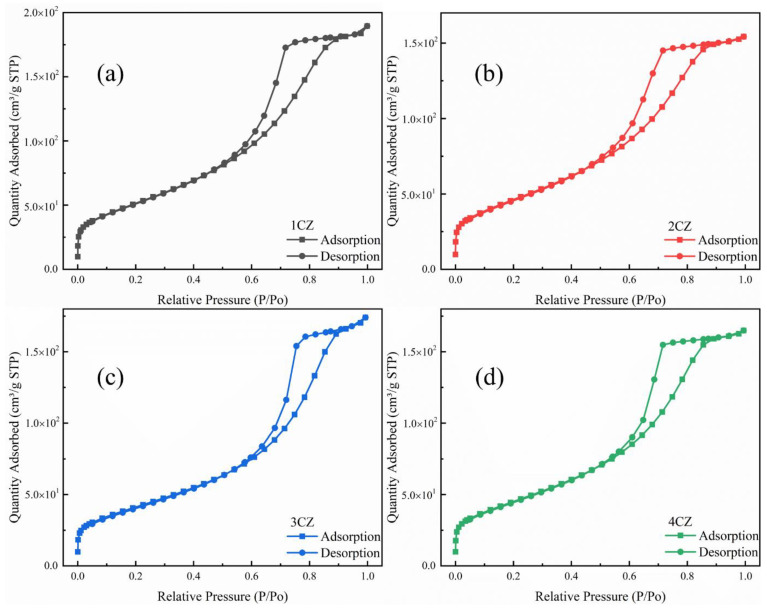
Isotherm linear plot for the CZ nanoparticles, (**a**) 1CZ; (**b**) 2CZ; (**c**) 3CZ; (**d**) 4CZ.

The isotherm linear plot for the CZ samples are shown in Figure 8. According to the classification of physical adsorption isotherms proposed by IUPAC, all samples belong to type IV adsorption isotherm. Type IV isotherms are generated by mesoporous solids. The typical characteristic is that the adsorption curve of the isotherm is inconsistent with the desorption curve, and hysteresis loops can be observed [42]. 

**Figure 9:**
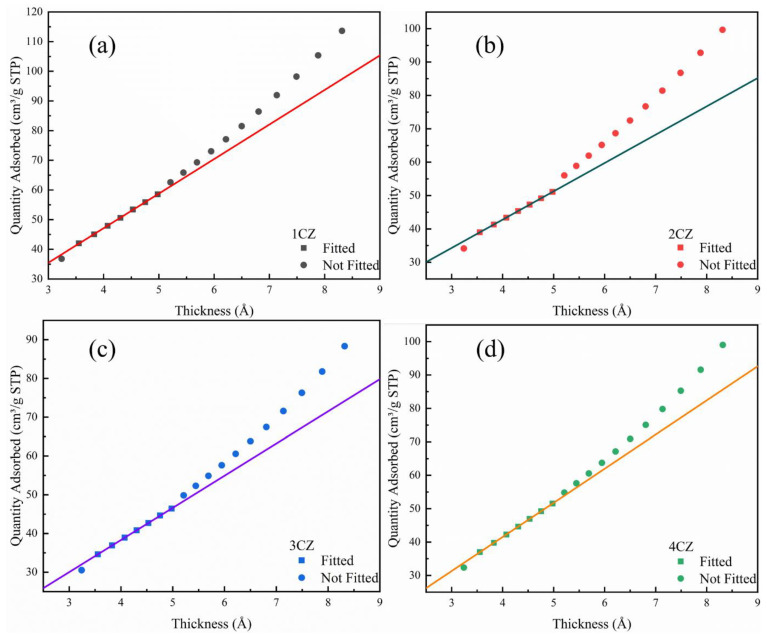
t-Plot for the CZ nanoparticles, (**a**) 1CZ; (**b**) 2CZ; (**c**) 3CZ; (**d**) 4CZ.

The t-plot method separates micropore from multilayer adsorption and the micropore analysis, based on the work of Dubinin and Radushkevich (DR) [43]. We applied the t-plot method, fitting the linear range in the t-plot, as shown in Figure 9. As a standard reference t-curve, we used the relation proposed by Harkins and Jura for calculating the micropore volume from the intercept [44], and the BJH method was used to analyze the volume of mesopores [45], as listed in Table 4. From the analysis result of the pore volume, the samples were mainly mesoporous.

**Figure 10:**
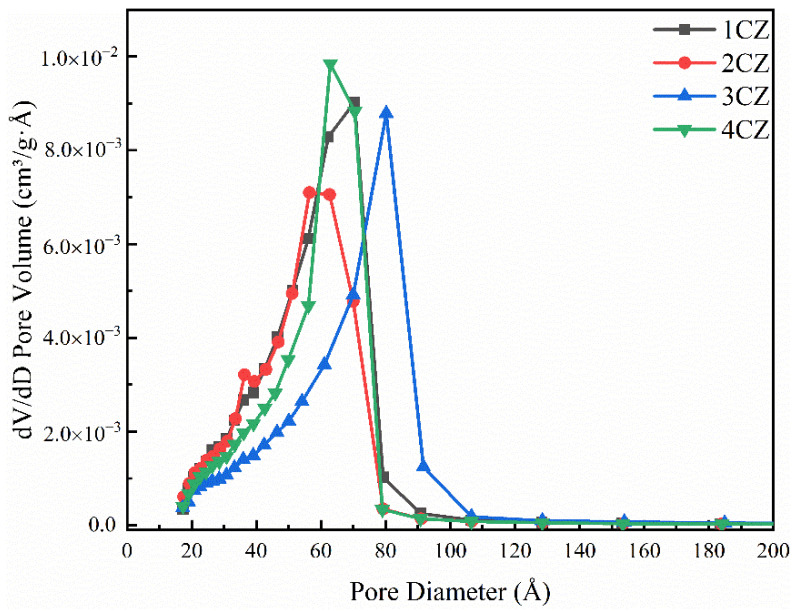
BJH Desorption dV/dD Pore Volume Distribution for the CZ nanoparticles.

Note that the BJH desorption curve was used to determine the distribution curve of pore volume in order of pore size, as shown in Figure 10. The average pore size of all samples ranged from 50 to 80 nm. The adsorption isotherms of CZ samples all had hysteresis rings. In this region, the amount of adsorption during desorption is always greater than that during adsorption under the same pressure. This phenomenon can be explained as follows: adsorption is caused by multimolecular adsorption on the pore wall and coagulation in the pore, while desorption is only caused by capillary desorption. During adsorption, multimolecular adsorption first occurs and, only when the adsorption layer on the pore wall is thick enough, condensation can occur, while in desorption, only evaporation occurs in the capillary. Therefore, in order to obtain the distribution curve of pore volume in order of pore size, the desorption curve should be used instead of the adsorption curve.

### 3.7. Oxygen Storage Capacity (OSC) Test

Oxygen pulse adsorption technology was used to test the oxygen storage capacity of the sample. The oxygen storage (OSC) test results for the CE sample are shown in Table 5. The oxygen storage of the sample increased from 433.7 μmol/g to 631.1 μmol/g when the Ce content increased from 10% to 40%. Zr^4+^ is a hexadecimal coordination, and Ce^4+^ is an octet coordination. After forming the ZrO_2_-CeO_2_ solid solution, Ce^4+^ enters the ZrO_2_ lattice to generate a large number of oxygen vacancies, which increases the activity of oxygen in the bulk phase and lowers the temperature of the redox reaction for CeO_2_. At moderate temperature, the Ce^3+^/Ce^4+^ redox reaction occurs not only at the oxygen defect sites on the surface of CeO_2_, but also in bulk CeO_2_, which results in a high oxygen storage capacity for the solid solution.

### 3.8. H_2_-TPR Test

**Figure 11:**
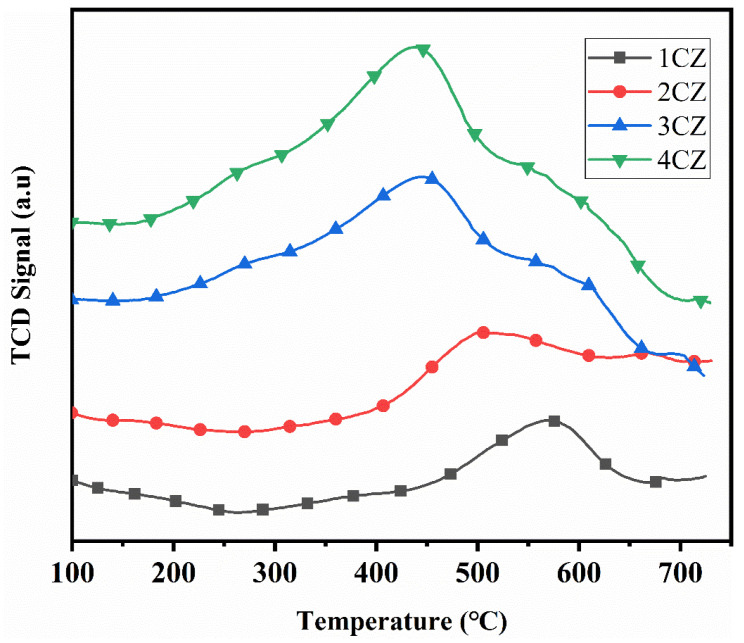
H_2_-TPR profiles for the CZ nanoparticles.

Figure 11 shows the H_2_-TPR test results for CZ samples. The four samples all show an obvious reduction peak at 300–700 °C, which indicates the reduction of CeO_2_ to Ce_2_O_3_ [46]. When the Ce:Zr ratios were 1:9 (1CZ) and 2:8 (2CZ), the reduction peak appeared between 500 and 600 °C. With an increase in the Ce:Zr ratio, the peak shifted towards low temperature. When the Ce:Zr ratios were 3:7 (3CZ) and 4:6 (4CZ), the reduction peak appeared in the range of 400–500 °C, indicating that the activation energy for the Ce^4+^/Ce^3+^ transformation decreases with increasing Ce:Zr ratio. This is because when the doping amount of Ce increases, the degree of lattice distortion due to ion doping increases, resulting in a large number of lattice oxygen defects and an improvement in the mobility of oxygen negative ions. This process promotes the transformation between Ce^4+^ and Ce^3+^.

## 4. Conclusions

A novel process for preparing CeO_2_-ZrO_2_ nanoparticles using a continuous hydrothermal flow synthesis (CHFS) system with a confined jet mixer (CJM) reactor is presented. Supercritical water (723 K, 24 MPa) was employed to prompt precursor solution hydrolysis and dehydration, resulting in CeO_2_-ZrO_2_ precipitation. A series of CeO_2_-ZrO_2_ complex oxides with different Ce/Zr ratios were successfully prepared by regulating the salt molar ratio of the precursor.

The FTIR spectra, Raman spectra and XRD results indicate that the obtained CeO_2_-ZrO_2_ samples were mixtures of tetragonal and cubic phases. TEM images showed that all CZ samples had high crystallinity, a uniform particle shape and narrow particle size distribution. The d-spacing values obtained by TEM were in good agreement with the reference data. BET results showed that the prepared CZ samples all had large specific surface areas, while the amount of Ce added had no great influence on the BET surface area. The isotherm linear plots showed all samples belonged to type IV adsorption isotherm. T-plot and BJH methods were used to determine the volume of micropore and mesopores. The BJH desorption curve was used to determine the pore size distribution. Oxygen pulse adsorption technology was used to test the oxygen storage capacity of the sample. The oxygen storage for the sample increased from 433.7 μmol/g to 631.1 μmol/g when the amount of Ce doping increased from 10% to 40%. The redox capacity of the CZ material was determined by a H_2_ temperature-programmed reduction test. An obvious reduction peak at 300–700 °C was observed in all samples, indicating the reduction of CeO_2_ to Ce_2_O_3_. With an increase in the Ce:Zr ratio, the reduction peak shifts towards low temperature.

## Figures and Tables

**Table 1:** CZ precursor solution preparation table.

	1CZ	2CZ	3CZ	4CZ
ZrO(NO_3_)_2_·xH_2_O (g)	20.8107	18.4984	16.1861	13.8738
Ce(NO_3_)_3_·6H_2_O (g)	4.3422	8.6844	13.0266	17.3688
Ce: Zr (molar ratio)	1:9	2:8	3:7	4:6

**Table 2:** Composition analyses of the obtained CZ samples.

Ce:Zr (Molar Ratio)	1CZ	2CZ	3CZ	4CZ
EDS	1.76:18.72	3.43:15.46	5.71:14.61	6.84:11.26
ICP-AES	2.16:20.47	4.61:19.4	6.22:15.51	7.69:12.54

**Table 3:** BET data for the CZ nanoparticles.

Date	1CZ	2CZ	3CZ	4CZ
BET surface area (m^2^/g)	147.7	164.4	153.6	161.1
C	92.75	83.88	83.07	96.14
Correlation coefficient	0.9999539	0.9999827	0.9999418	0.9999625

**Table 4:** t-Plot micropore volume and BJH mesopore volume for the CZ nanoparticles.

Volume (cm^3^/g)	1CZ	2CZ	3CZ	4CZ
t-Plot	0.005626	0.006665	0.002708	0.003913
BJH	0.289700	0.245361	0.273827	0.260834

**Table 5:** OSC data for CZ nanoparticles.

Samples	1CZ	2CZ	3CZ	4CZ
OSC (μmol/g)	433.7	495.6	535.7	631.1

## Data Availability

Not applicable.
